# Paravertebral block for analgesia following excision of osteochondroma of the scapula: A case report

**DOI:** 10.7555/JBR.37.20230048

**Published:** 2023-09-10

**Authors:** Deepthi L. Penta, Usha Saldanha, Hong Liu

**Affiliations:** Department of Anesthesiology and Pain Medicine, University of California, Davis Health, Sacramento, CA 95817, USA

**Keywords:** paravertebral block, analgesia, scapular osteochondroma

## Abstract

Scapular surgery has mainly been studied in the setting of fractures; regional anesthesia can be utilized as part of a multimodal analgesia regimen for postoperative pain relief. Previous studies are limited to scapular fracture pain. The available literature supports the use of various types of nerve blocks and even combinations of different blocks, of which the paravertebral nerve block is one such block that has been effective. We present a case of a patient undergoing excision of a scapular osteochondroma who received a single-shot paravertebral nerve block after surgery with an effective analgesia.

## Introduction

Osteochondromas are benign growths of the bone. They occur as solitary tumors or as a genetic neoplastic syndrome known as hereditary multiple osteochondromas. Common sites of the occurrence include the scapula, humerus, radius, femur, tibia, and fibula. The patients may select surgical treatment to mitigate the symptoms of pain, limited range of motion, nerve compression, growth disturbance, or cosmetic reasons that are usually resolved with surgery, and the associated morbidity of surgery is low ^[ 1] ^. The literature available about scapular surgery in the context of fractures is uncommon, and these patients were usually managed conservatively. When surgery is pursued, general anesthesia with or without regional anesthesia is chosen ^[ 2] ^. Overall, scapular fractures account for only about 0.4% to 1% of all fractures; thus, the literature on regional anesthetic techniques in the setting of scapular fractures is limited ^[ 3] ^. We report a case of a patient with symptomatic osteochondroma of the scapula. This case report is the first to describe the use of regional anesthesia to provide analgesia specifically for the surgical excision of a scapular osteochondroma. The institutional review board at the University of California, Davis does not require a formal approval for case reports. Permission was obtained, and a written informed consent from the patient was presented in this case.

## Case description

**Figure 1:**
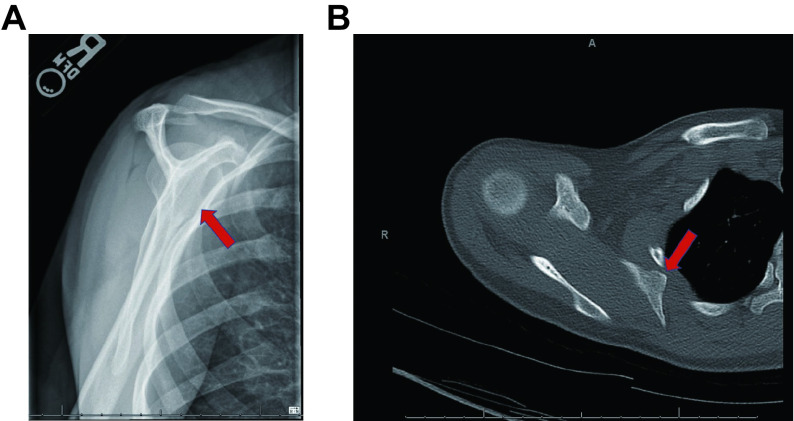
The anatomic location of the tumor.

A 26-year-old female, who had a past medical history significant for hereditary multiple exostoses, multiple hereditary osteochondromas, and functional neurological symptom disorder, presented to the orthopedic oncology service with ongoing right shoulder pain associated with a snapping sensation of the scapula ( ***Fig. 1***). The patient had a significant past surgical history that included prior excision of multiple lower extremity osteochondromas starting in 2007. Of note, surgical resection of the left scapula and proximal humerus osteochondroma occurred during the prior year with post-surgical placement of an interscalene block. Because of persistent right upper extremity pain with movement and a failed conservative therapy, the surgical team planned for an excision of the scapular body osteochondroma with local infiltration. Of note, the patient had significant drug allergies to multiple pain medications, including dilaudid, morphine, oxycodone, and tramadol, that cause hives and dyspnea. A local anesthetic infiltration by the surgical team consisting of 25 mL of 0.25% bupivacaine was injected into the skin and deep muscle. Prior to block placement in the post-anesthesia care unit, the patient reported a pain score of 9/10 on the numeric rating scale.

**Figure 2:**
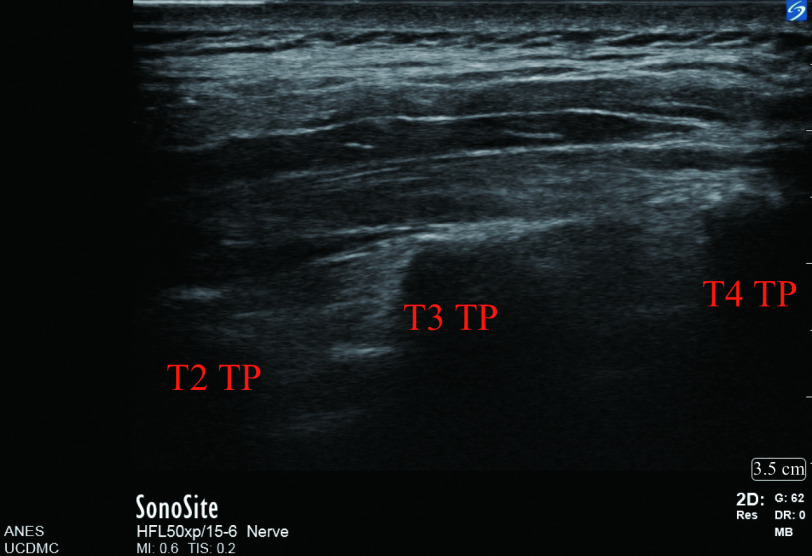
Ultrasound image of the paravertebral block.

The patient was positioned sitting upright, with the standard placement of American Society of Anesthesiology monitors. The skin on the back was cleaned with chlorhexidine gluconate before applying a sterile drape. A linear 15-6 MHz, HFL50, X-Porte (Sonosite, Bothell, WA, USA) ultrasound probe was placed in a parasagittal orientation with visualization of the paravertebral space between the second and third thoracic vertebrae. A 17-G 3.5-inch Tuohy needle was inserted between the second and third transverse processes using an in-plane technique with continuous needle visualization ( ***Fig. 2***). A total of 20 mL of 0.25% ropivacaine with 50 μg epinephrine was incrementally injected with ease; a frequent aspiration was performed to avoid intravascular injection. The patient tolerated the procedure well without complications.

About 30 min after the block, her pain improved significantly to a three out of 10 score. For the next 10 h, her pain ranged between three and six on the numerical rating scale with an analgesic duration of 12 h. During this time, she received only two doses of 25 μg intravenous fentanyl in addition to other scheduled non-opioid medications. Twenty-four hours after block placement, the patient reported a pain score of eight out of 10, which was overall well controlled with multimodal analgesia, including scheduled Tylenol, Toradol, and ketamine. On postoperative day 1, she was able to participate in physical therapy and was discharged home with Tylenol and Motrin for pain control.

## Discussion

**Figure 3:**
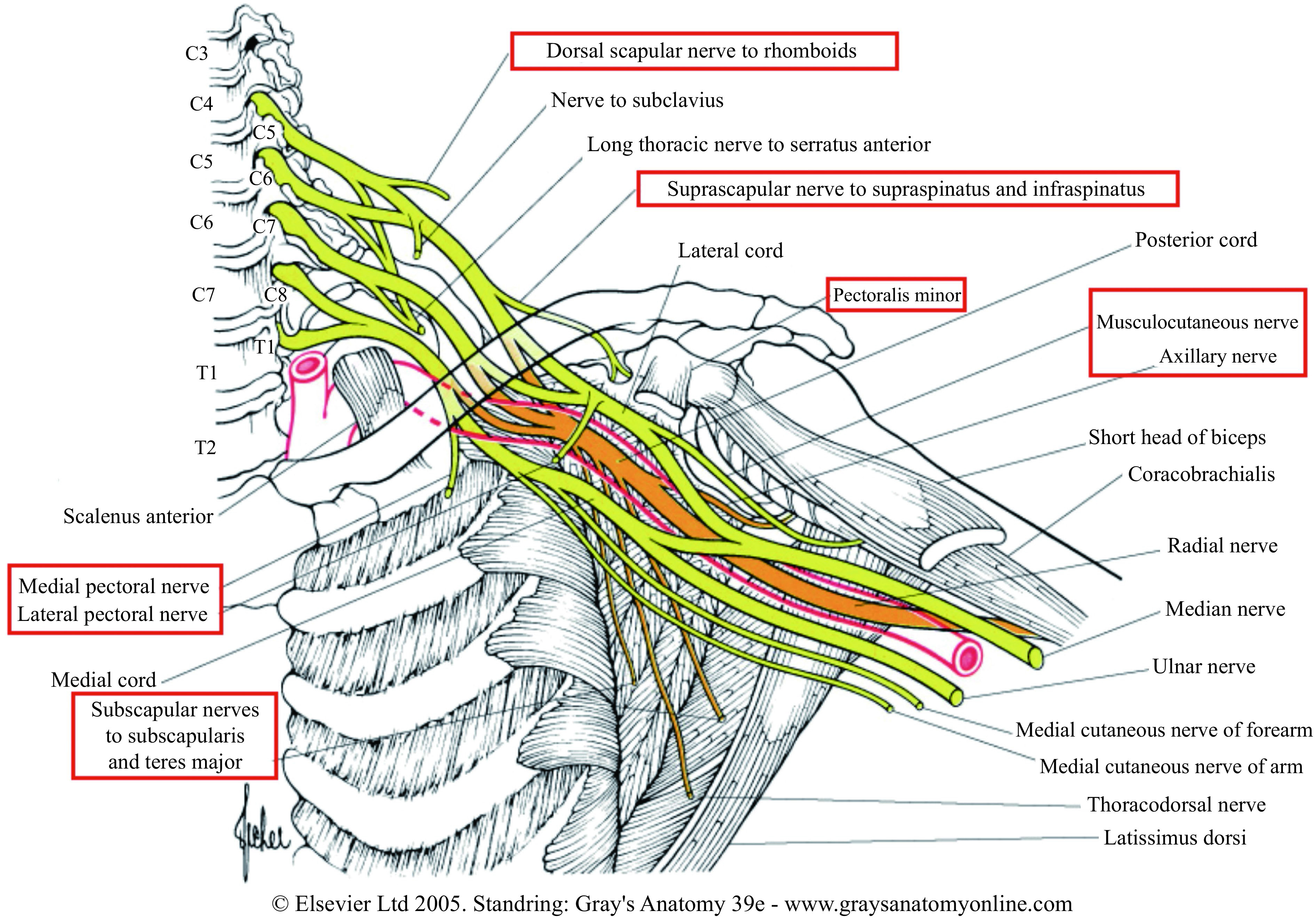
Schematic of brachial plexus including scapular innervation.

The scapula is a triangular and flat bone that stabilizes the shoulder girdle and plays an important role in the upper limb function. The innervation of the scapular region is quite complex, involving the brachial plexus, superficial cervical plexus, and intercostal nerves ^[ 4] ^. More specifically, the scapula is innervated by the dorsal scapular (C5), upper and lower subscapular (C5-6), and suprascapular nerves (C5-6) ^[ 5]^ ( ***Fig. 3***). The paravertebral nerve block involves a local anesthetic injection into a space adjacent to the vertebral bodies and lateral to where spinal roots exit the intervertebral foramina. These nerve roots divide into dorsal and ventral rami within this space. Each space communicates with adjacent spaces, allowing the spread to multiple contiguous dermatomes ^[ 6] ^. Because the paravertebral nerve block covers both the ventral and dorsal rami, it produces a dense block with both motor and sensory blockades ^[ 5] ^. Additionally, the thoracic paravertebral space is continuous with the intercostal space laterally ^[ 6] ^. Thus, this block is useful for scapular surgeries as it blocks the intercostal nerves supplying the scapular dermatome ^[ 4] ^. This is the likely mechanism for analgesia in the described case.

The use of a paravertebral block for scapular fracture has been reported with success previously. For example, Curran and colleagues described the use of a continuous paravertebral nerve block for a comminuted scapular body fracture being managed nonoperatively, and reported that the placement of a T2-T3 paravertebral catheter resulted in improved pain scores and the involvement in physical therapy ^[ 3] ^.

In the current case, the patient had a previous resection of osteochondroma from the scapula and proximal humerus. Proximal humerus pain was reported as minimal; however, the patient had significant scapular pain postoperatively. Placement of the paravertebral single shot block after the subsequent osteochondroma resection appeared to markedly alleviate post-surgical pain for 12 h. This was especially useful in a patient with multiple opioid insensitivities and chronic pain. Catheter placement could have been considered due to a history of previous difficult pain control and insensitivity to opioids, and several different regional techniques have been described for scapular surgery ( ***Table 1***).

**Table 1:** Different regional techniques for scapular surgery

Regional technique	Mechanism	Anatomy/Innervation	Reference
Combination block ("dual injection approach"): 1) Interscalene/selective supraclavicular block 2) Erector spinae plane at T2	Analgesia *via* coverage of various dermatomes, osteotomes, and myotomes	1) Dorsal scapular, suprascapular, subscapular, supraclavicular, axillary, lateral/medial pectoral, and musculocutaneous nerves 2) Dorsal rami of C6-T5 (skin over the back)	[ 2]
Combination block ("triple injection approach"): 1) Selective upper trunk/supraclavicular block 2) Subclavian perivascular block/suprascapular block 3) Erector spinae plane at T2	Analgesia or surgical anesthesia *via* coverage of various dermatomes, osteotomes, and myotomes (covers variations of brachial plexus)	1) Dorsal scapular, suprascapular, supraclavicular nerves 2) Subscapular, axillary, lateral/medial pectoral, and musculocutaneous nerves 3) Dorsal rami of C6-T5 (skin over the back)	[ 2]
Suprascapular block	Blocks sensation to the scapula	Upper trunk of brachial plexus (C4-C6 nerve roots)	[ 7]

In conclusion, we have illustrated a case in which a paravertebral single-shot nerve block was successfully used for analgesia after the excision of a scapular osteochondroma in a patient who was unable to tolerate multiple intravenous pain medications. The literature regarding scapular surgeries and regional anesthesia for these procedures is available but scarce, and most reports are in the context of scapular fractures specifically. Because of the complex innervation of the scapula, regional techniques combining different blocks tailored to the specific procedure may be useful.
